# Longitudinally engineered metasurfaces for 3D vectorial holography

**DOI:** 10.1038/s41377-025-02158-5

**Published:** 2026-01-03

**Authors:** Le Tan, Pengcheng Huo, Peicheng Lin, Yongze Ren, Haocun Qi, Lizhi Fang, Yilin Wang, Junfei Ou, Yanqing Lu, Ting Xu

**Affiliations:** 1 National Laboratory of Solid-State Microstructures, and Collaborative Innovation Center of Advanced Microstructures, Nanjing University, Nanjing, China; 2 School of Electronic Science and Engineering, Nanjing University, Nanjing, China; 3 College of Engineering and Applied Sciences, and Key Laboratory of Intelligent Optical Sensing and Manipulation, Nanjing University, Nanjing, China; 4 School of Materials Engineering, Jiangsu University of Technology, Changzhou, China

**Keywords:** Metamaterials, Sub-wavelength optics

## Abstract

The ability to precisely generate and manipulate three-dimensional (3D) vectorial optical fields is crucial for advancing applications in volumetric displays, secure data encoding, and optical information processing. However, conventional holographic techniques generally lack the capability to simultaneously control both light intensity and polarization within a volumetric region, thereby limiting the full realization of complex 3D vectorial light fields. Here, we present a metasurface-based platform for 3D vectorial holography that enables independent and programmable control over axial intensity and polarization profiles within structured beam arrays. By decomposing complex volumetric holographic targets into a dense array of non-diffracting beams—each governed by a tailored longitudinal response function—we achieve broadband, high-fidelity reconstruction of vectorial light fields encoded in both spatial intensity and polarization domains. Moreover, we demonstrate a vectorial encryption scheme that exploits the combined axial intensity and polarization degrees of freedom to realize secure, key-based optical information encoding. This approach provides a compact, integrable, and scalable solution for 3D vectorial holographic projection and volumetric vector beam shaping, offering a versatile platform for high-capacity optical storage, secure communication, and emerging quantum photonic technologies.

## Introduction

Over the past few decades, the demand for precise generation and manipulation of complex three-dimensional (3D) optical fields has grown significantly, driven by applications in optical data storage^1–3^, secure information encryption^4–7^, laser beam shaping^8,9^, augmented/virtual reality^10–12^, advanced optical trapping^13,14^, and volumetric microscopic imaging^15–17^. Holography, as a fundamental technique for recording and reconstructing arbitrary wavefronts, offers unique advantages for 3D visualization and volumetric light-field control^18–20^. Nevertheless, conventional holographic approaches, including those based on spatial light modulators (SLMs), digital micromirror devices (DMDs), or bulky volumetric media, often suffer from limitations in system integration, efficiency, and their ability to fully exploit the vectorial nature of light^21–24^. These challenges become particularly critical when simultaneous modulation of both intensity and polarization within a volumetric region is required, thereby preventing conventional methods from achieving true 3D vectorial holography.

Metasurfaces, which are ultrathin optical devices composed of subwavelength scatterer arrays, have emerged as a powerful platform for precise control of multiple light-field parameters, including phase, amplitude, polarization, and frequency^25–36^. This paradigm shift has enabled the development of compact and efficient holographic devices with unprecedented flexibility. Most metasurface-based holograms reported to date have primarily focused on phase and amplitude encoding for scalar field modulation, with some extensions toward two-dimensional (2D) vectorial holography. Building upon Fresnel and Fourier holography, both 2D and 3D scalar holography have been demonstrated, ranging from simple image projection to volumetric reconstructions^37–42^. More recently, efforts have been made to simultaneously control intensity and polarization, representing an important step toward vectorial holography^43–48^. However, these demonstrations remain largely restricted to 2D vectorial holography confined to a single plane. Achieving full 3D vectorial holography, which requires coordinated modulation of amplitude, depth, and polarization across an entire volume, remains a formidable challenge that calls for novel design strategies extending beyond conventional metasurface principles.

In this work, we present a metasurface-based platform for 3D vectorial holography, wherein the target volumetric image is decomposed into an array of structured beams with independently programmable axial intensity and polarization profiles. The longitudinal evolution of each beam is determined by a tailored response function, enabling the construction of high-density beam arrays with spatially varying vectorial properties. As a proof of concept, we experimentally implement multiple metasurfaces that encode broadband axial intensity shaping together with independently tunable polarization states. Leveraging this capability, we further demonstrate a polarization- and depth-encoded encryption scheme, in which information is securely embedded within a 3D vectorial light field and can only be decrypted using key-matched polarization and axial parameters. These results establish a compact, scalable, and integrable framework for 3D vectorial holography, opening new opportunities for high-capacity optical communication, secure data storage, and quantum information processing.

## Results

### Theory and principle

**Fig. 1:**
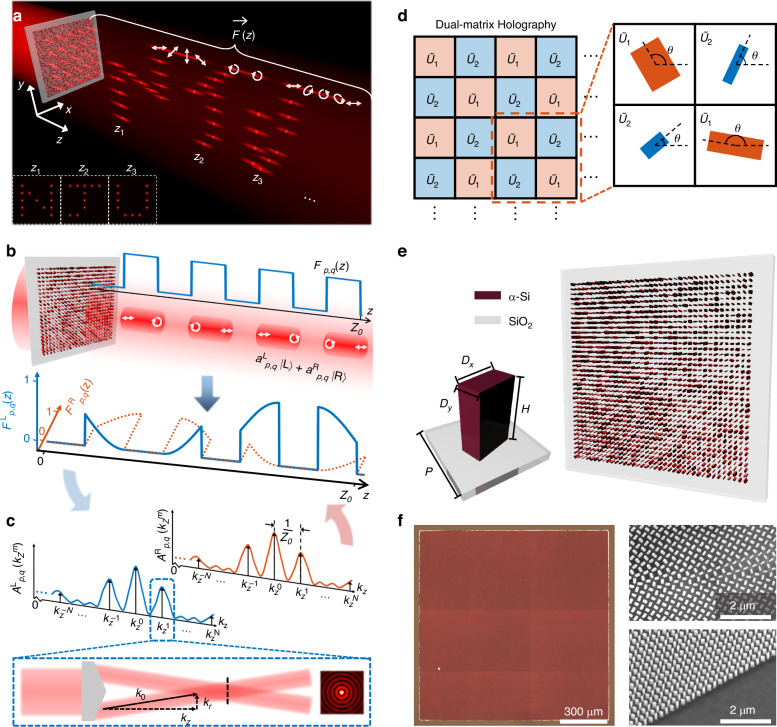
**Design principles of 3D vectorial holography using longitudinally tunable beam arrays.** **a** Schematic of the 3D vectorial holography based on a *z*-dependent array of structured beams, with independently controlled axial intensity and polarization profiles. **b** Construction of the target longitudinal response function, composed of the axial intensity envelope and polarization trajectory. The function can be decomposed into complex value functions in the LCP and RCP bases. **c** Modulation strategy based on a superposition of 2 *N* + 1 Bessel functions with uniform spacing in ${k}_{z}$-space. **d** Schematic of the metasurface architecture, composed of rectangular-shaped *α*-Si nanopillars on a SiO_2_ substrate. **e** Top-view schematic of the dual-matrix holographic metasurface, where nanopillar dimensions and orientation encode the desired unitary Jones matrix at each pixel. **f** Optical photographs and SEM images (top and tilted views) of the fabricated metasurfaces, designed for the independent axial intensity and polarization manipulation

To achieve holographic projections of 3D vectorial light fields that simultaneously embed both depth and polarization information, we decompose the target field into an array of quasi-non-diffracting beams, each with independently engineered longitudinal intensity and polarization profiles, as illustrated in Fig. 1a. To construct the desired volumetric 3D vectorial holography, each beam within the array is assigned a unique longitudinal response function$\,{\vec{F}}_{p,q}\left(z\right)$ that governs its evolution along the optical axis (*z*), where *p* and *q* denote the row and column indices of the beam in the array, respectively. As shown in Fig. 1b, the longitudinal response function comprises a scalar function ${F}_{p,q}\left(z\right)$ defining the axial intensity profile, and a Jones vectorial function that describes the spatially varying polarization state along the propagation axis. In principle, the vectorial component can be represented as a linear coherent superposition of two orthogonal circular polarization basis states with *z*-dependent complex weighting coefficients ${a}_{p,q}^{{\rm{L}}}\left(z\right)$ and ${a}_{p,q}^{{\rm{R}}}(z)$, respectively. Through the orthogonal circular polarization decomposition, the complete longitudinal response function can be expressed as

1 $${\vec{F}}_{p,q}(z)={F}_{p,q}(z)[{a}_{p,q}^{L}(z){|\; L}\rangle +{a}_{p,q}^{R}(z){|R}\rangle ]$$

where $${|L}\rangle =\frac{1}{\sqrt{2}}\left[\begin{array}{c}1\\ i\end{array}\right]$$ and $${|L}\rangle =\frac{1}{\sqrt{2}}\left[\begin{array}{c}1\\ -i\end{array}\right]$$ denote the unit vectors for left- and right-circular polarization (LCP and RCP), respectively. The weighting coefficients satisfy the normalization condition ${|{a}_{p,q}^{L}\left(z\right)|}^{2}+{|{a}_{p,q}^{R}\left(z\right)|}^{2}=1$. Therefore, the function ${\vec{F}}_{p,q}\left(z\right)$ can be decomposed into two spin-dependent complex value functions, ${F}_{p,q}^{{\rm{L}}}\left(z\right)={F}_{p,q}\left(z\right){a}_{p,q}^{{\rm{L}}}\left(z\right)$ and ${F}_{p,q}^{{\rm{R}}}\left(z\right)={F}_{p,q}\left(z\right){a}_{p,q}^{{\rm{R}}}$, corresponding to two orthogonal LCP and RCP channels, respectively.

To realize these longitudinal profiles, we employ a Fourier-like synthesis method, in which each beam is constructed as a coherent superposition of discrete Bessel beams with a series of longitudinal wavevectors evenly spaced in the *k*_*z*_-domain, as illustrated in Fig. 1c. The complex weighting coefficients of these Bessel components are determined by projecting the desired longitudinal response functions onto the basic exponential functions as (see Supplementary Section 1 for details)

2 $${A}_{p,q,m}^{{\rm{L}}/{\rm{R}}}=\frac{1}{{Z}_{0}}{\int }_{0}^{{Z}_{0}}{F}_{p,q}^{{\rm{L}}/{\rm{R}}}\left(z\right){e}^{-i\frac{2\pi }{{Z}_{0}}{mz}}{\rm{d}}z$$

where ${A}_{p,q,m}^{{\rm{L}}/{\rm{R}}}$ are complex-valued coefficients that control the amplitude and phase of the *m*-th Bessel beam component in the LCP or RCP channels, and *Z*_0_ defines the axial extent of the modulated beam array. The discrete spacing Δ*k*_*z*_ = 2*π*/*Z*_0_ of the superimposed Bessel components further ensures mutual orthogonality of the Bessel basis components over the defined axial range. Hence, the complete generated 3D vectorial light field can be expressed as the coherent summation of all the spin-dependent Bessel functions across the full beam array, which can be written as

3 $$\vec{E}(\boldsymbol{\rho} ,z)=\mathop{\sum}\limits_{p,q}\mathop{\sum }\limits_{m=-N}^{N}{J}_{0}({k}_{\rho }^{m}\Vert \boldsymbol{\rho} -{\boldsymbol{\rho} }_{p,q}\Vert ){e}^{{{ik}}_{z}^{m}z}\,\cdot \,\left[{A}_{p,q,m}^{L}{|L}\rangle +{A}_{p,q,m}^{R}{|R}\rangle \right]$$

where ${J}_{0}\left(\cdot \right)$ refers to the zeroth-order Bessel function of the first kind, ${{\boldsymbol{\rho }}}_{p,q}$ refers to the lateral displacement of the beam, and the transverse and longitudinal wavevectors ${k}_{\rho }^{m}$ and ${k}_{z}^{m}$ satisfy the dispersion relation ${\left({k}_{\rho }^{m}\right)}^{2}+{\left({k}_{z}^{m}\right)}^{2}={\left({k}_{0}\right)}^{2}$, where *k*_*0*_ is the wavevector in free space. Substituting *z* = 0 into Eq. (3) yields the initial complex field distributions at the plane of the metasurface for both orthogonal polarization components. Based on the profiles in the two polarization channels, the Jones matrix profile of the metasurface ${\widetilde{J}}_{{\rm{meta}}}\left(\rho ,\varphi \right)$ can be determined to realize 3D holographic projection with full vectorial field manipulation, after deciding the polarization state of the incident light.

To experimentally realize the desired vectorial light field projection via the structured beam array, the metasurface must be engineered to support subwavelength modulation of phase, amplitude, and polarization. As illustrated in Fig. 1d, the designed metasurface comprises an array of rectangular-shaped amorphous silicon (*α*-Si) nanopillars with a height of 400 nm and a lattice period of 250 nm, fabricated on a fused silica substrate. Furthermore, the complex-valued Jones matrix profile ${\widetilde{J}}_{{\rm{meta}}}\left(\rho ,\varphi \right)$ is converted into a physically realizable unitary matrix profile ${\widetilde{U}}_{{\rm{meta}}}\left(\rho ,\varphi \right)$ by leveraging a dual-matrix holography technique (see Supplementary Section 3 for details). The unitary matrix profile can finally be implemented by the metasurface according to the following equation

4 $${\widetilde{U}}_{{\rm{meta}}}\left(\rho ,\varphi \right)=\widetilde{R}\left(-\theta \left(\rho ,\varphi \right)\right)\left[\begin{array}{cc}{t}_{x}{e}^{i{\phi }_{x}\left(\rho ,\varphi \right)} & 0\\ 0 & {t}_{y}{e}^{i{\phi }_{y}\left(\rho ,\varphi \right)}\end{array}\right]\widetilde{R}\left(\theta \left(\rho ,\varphi \right)\right)$$

where $\widetilde{R}\left(\theta \right)$ is the rotation matrix accounting for the local orientation *θ*(*ρ*,*φ*) of the nanopillar, and *t*_*x*_, *t*_*y*_, *ϕ*_*x*_, and *ϕ*_*y*_ define amplitude and phase values of the complex transmission coefficients for linearly polarized light along the *x* and *y* directions, respectively.

As schematically shown in Fig. 1e, the geometry (*D*_*x*_, *D*_*y*_) and in-plane orientation *θ* of each nanopillar are independently tailored to implement the designed matrix profile, according to the dual matrix holography. This design enables subwavelength control over both phase and polarization at each metasurface pixel. Figure 1f presents the optical micrograph and scanning electron microscope (SEM) images of the fabricated metasurface, revealing the high-fidelity nanopillar array structure. The fabricated metasurface has a lateral size of 1.2 mm and supports a designed axial modulation depth of 3 mm. Detailed fabrication procedures are provided in the Methods section.

### Broadband holography with axial intensity control

We first demonstrate the capability of the metasurface to modulate the longitudinal intensity profile while preserving a constant polarization state along the propagation direction. This proof-of-concept experiment validates longitudinal intensity shaping, a key requirement for constructing 3D holographic projections. In the setup, the metasurface is illuminated by a coherent beam derived from a supercontinuum laser source coupled with an acousto-optic tunable filter for wavelength selection. A polarization control device placed before the metasurface prepares the incident state, while a second device after the metasurface analyzes the output polarization of the transmitted beams. The metasurface is mounted on a motorized translation stage, enabling precise measurements of field distributions at multiple axial positions along the *z*-axis.

**Fig. 2:**
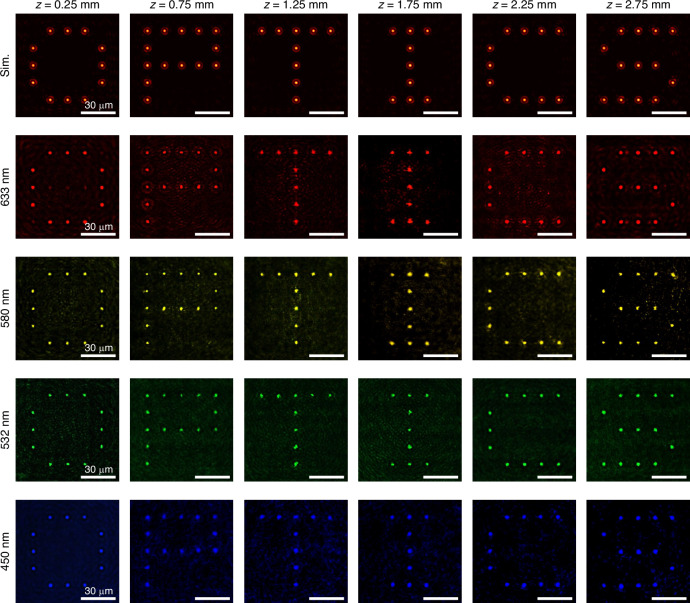
**Broadband reconstruction of volumetric intensity patterns using the beam array with longitudinally varying intensity profiles and constant polarization.** The transverse intensity distributions of a 5 × 5 beam array forming sequential letters (“O,” “P,” “T,” “I,” “C,” “S”) along the *z*-axis, ranging from *z* = 0.25 mm to *z* = 2.75 mm. The top row refers to the simulated transverse intensity distributions of the array at the designed axial locations. The second to fifth rows refer to the experimentally measured results of the corresponding transverse intensity distributions under illumination at wavelengths of 633, 580, 532, and 450 nm, respectively. Scale bars: 30 μm. All patterns exhibit high contrast, uniformity, and broadband consistency. The abbreviation “Sim” has the full name of “Simulation”

By independently controlling the longitudinal response of each beam in the array concerning the axial positions, a series of transverse intensity patterns is sequentially projected along the propagation axis within a well-defined 3D volume. The 5 × 5 Bessel beam array is designed to successively project the letters “O,” “P,” “T,” “I,” “C,” and “S” at different axial positions *z* = 0.25 mm, 0.75 mm, 1.25 mm, 1.75 mm, 2.25 mm. The first row of Fig. 2 presents the simulated transverse intensity distributions at the target axial planes. The second to fifth rows present the experimentally measured intensity profiles under coherent illumination at wavelengths of 633 nm, 580 nm, 532 nm, and 450 nm, respectively. The brightened beams exhibit uniform intensity across each transverse plane, ensuring high-contrast and clearly distinguishable projections. Minor wavelength-dependent axial shifts, attributed to chromatic dispersion in the longitudinal wavevector spectrum, are observed. Nonetheless, the consistent reproduction of the designed patterns across a broad spectral range demonstrates the broadband robustness of the metasurface for 3D image projection.

### 3D vectorial field reconstruction

To demonstrate 3D vectorial holography with simultaneous control of intensity and polarization, we fabricated two metasurfaces, each with a lateral size of 1.2 mm and designed for modulation over an axial range of 3 mm. The experimental configuration is similar to that used in previous demonstrations, with additional polarization control elements incorporated. The first metasurface sequentially projects the letters “N” and “J” within two distinct axial regions, spanning *z* = 0.6–1.2 mm and *z* = 1.7–2.3 mm, respectively. Within each region, the beam intensity is shaped to follow a smooth cosine-type axial envelope. The second metasurface encodes four letters—“M,” “E,” “T,” and “A”—over *z* = 0.1-0.6 mm, 0.9-1.4 mm, 1.6-2.1 mm, and 2.4–2.9 mm, respectively, with binary-valued axial intensity functions defining the on-off switching of each symbol. The longitudinal intensity profile of each beam is independently engineered through the superposition of constituent Bessel modes, allowing precise axial positioning of the transverse patterns. In parallel, the longitudinal polarization states are programmed to evolve along customized trajectories on the Poincaré sphere. For these two metasurfaces, the trajectories are constrained to the equatorial plane, rotating in opposite directions under linearly polarized illumination—one completing a half counter-clockwise rotation, and the other a full clockwise rotation—thereby generating continuously varying longitudinal polarization states.

**Fig. 3:**
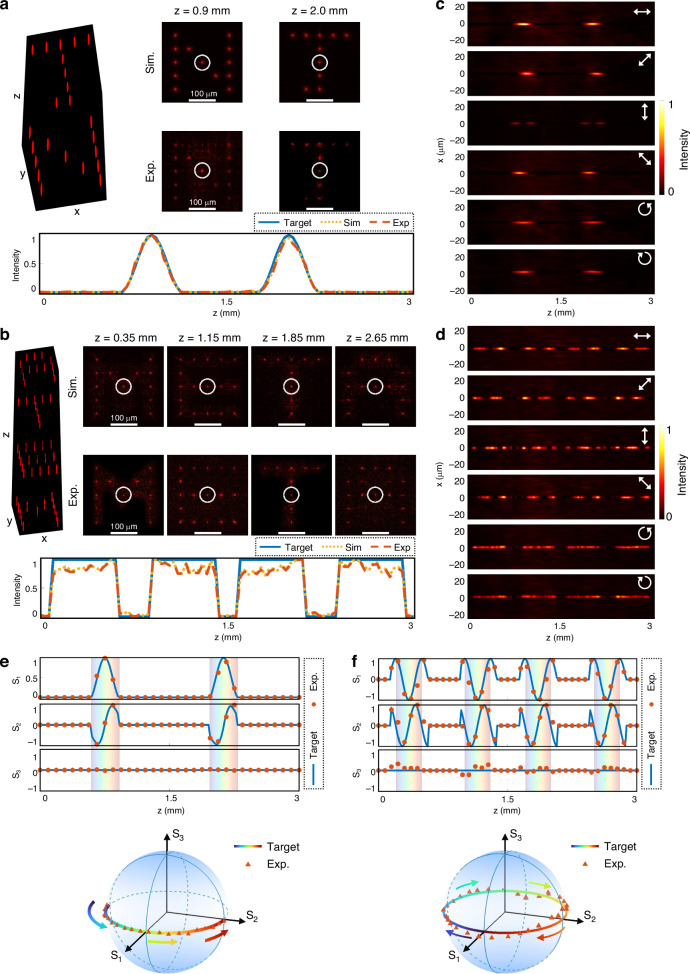
**Demonstration of the 3D vectorial holography using a beam array with different longitudinal intensity profiles and rotating linear polarization state.** **a**, **b** The simulated and the experimentally measured transverse intensity distributions at designated axial planes generated by (**a**) the first and (**b**) the second metasurface. Scale bars: 100 μm. The target axial intensity response functions (the blue lines), the simulated (the yellow dashed lines), and the experimentally measured intensity profiles (the red dashed lines) of the beam marked by the white circles are attached below. The abbreviation “Exp” has the full name of “Experiment”. **c**, **d** The experimentally measured intensity distributions around the center beams (as highlighted in (**a**, **b**)) in the *x*-*z* plane after transmission through different polarizers, which is depicted by the white arrow. **e**, **f** The target (the blue lines) and experimentally measured (the red dots) normalized Stokes parameters of the beam highlighted in (**a**, **b**) as functions of *z*. The Stokes trajectory on the Poincaré sphere is plotted based on Stokes vectors (*S*_1_, *S*_2_, *S*_3_)^*T*^, confirming continuous rotation of linear polarization

Figure 3a, b presents simulated and experimentally measured transverse intensity distributions at the target axial positions. The experimental results closely reproduce the simulated patterns across the axial range. To analyze intensity evolution in detail, a *z*-axis scan was performed, and the beam intensities highlighted by white circles were recorded. The corresponding envelopes, plotted beneath Fig. 3a, b, agree well with the designed profiles. Minor deviations observed in the second metasurface arise from the finite number of Bessel components (*N*) used in the superposition, as discussed in Eq. (3). Additional results, provided in Supplementary Section 6, further verify independent longitudinal modulation across the beam array, which is essential for constructing spatially varying 3D vectorial holograms.

To confirm the evolution of polarization states along the propagation axis, full Stokes polarimetry is carried out to calculate the normalized Stokes parameters *S₁*, *S₂*, and *S₃*. These parameters are derived using six intensity measurements taken through linear polarizers aligned along 0°, 90°, 45°, and 135°, and circular polarizers for right- and left-handed components (see Supplementary Section 7 for details): *S*_*1*_ = (*I*_0°_−*I*_90°_)/(*I*_0°_ + *I*_90°_), *S*_*2*_ = (*I*_45°_−*I*_135°_)/(*I*_45°_ + *I*_135°_), *S*_*3*_ = (*I*_RCP_−*I*_LCP_)/(*I*_RCP_ + *I*_LCP_). Figure 3c, d shows the intensity distributions of the beams in the *x-z* plane under these six analyzer settings. Based on these measurements, the normalized Stokes parameters along the beam center are reconstructed, and the corresponding polarization state evolution is also mapped on the Poincaré sphere, as shown in Fig. 3e, f. These plots clearly visualize the polarization trajectories, which match the target designs, confirming intended vectorial modulation along the propagation axis. The experimental results corroborate the simulated trajectories, demonstrating the platform’s precision and flexibility in simultaneously realizing longitudinally varying intensity profiles and polarization states.

**Fig. 4:**
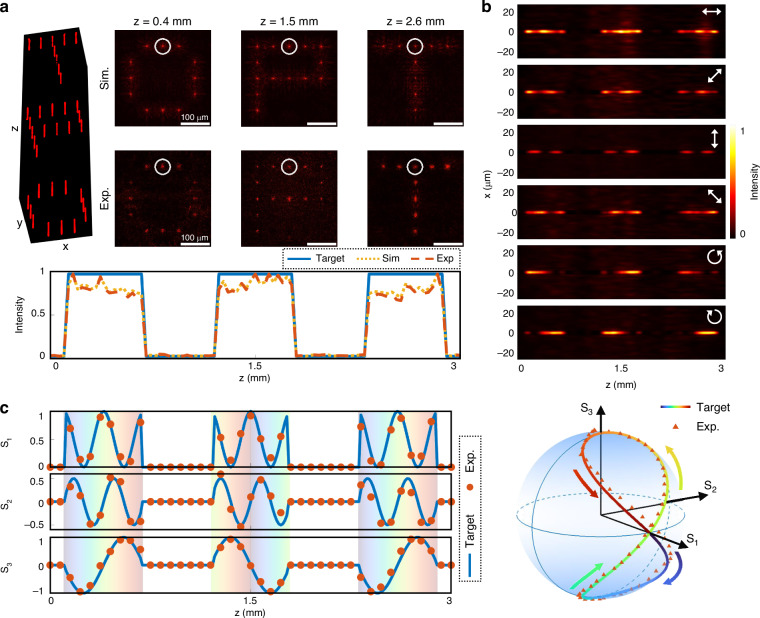
**Demonstration of the complex 3D vectorial holography using beam array with different longitudinal intensity profiles and helicity-varying polarization state.** **a** The simulated and the experimentally measured transverse intensity distributions at designated axial planes. Scale bars: 100 μm. The target axial intensity response function (the blue line), the simulated (the yellow dashed line), and the experimentally measured intensity profile (the red dashed line) of the beam marked by the white circles are attached below. **b** The experimentally measured intensity distributions around the beam highlighted in (**a**) in the *x*-*z* plane after transmission through different polarizers, which is depicted by the white arrow. **c** The target (the blue line) and experimentally measured (the red dots) normalized Stokes parameters of the beam highlighted in (**a**) as a function of *z*. The Stokes trajectory on the Poincaré sphere is plotted based on Stokes vectors (*S*_1_, *S*_2_, *S*_3_)^*T*^, revealing full helicity modulation, connecting the north and south poles of the sphere

To highlight more complex polarization modulation, we further designed a metasurface that introduces chirality evolution into the longitudinal polarization response. This metasurface projects the letters “O,” “P,” and “T” at *z* = 0.12–0.68 mm, 1.22–1.78 mm, and 2.32–2.88 mm, respectively, each defined by binary-valued axial intensity profiles. The simulated and measured transverse patterns are shown in Fig. 4a, where the recorded beam intensities closely follow the target envelopes. Full polarization analysis is also performed along the propagation axis. The beam intensities, filtered through different polarization bases and recorded in the *x-z* plane, are shown in Fig. 4b. The corresponding Stokes parameters are calculated and plotted in Fig. 4c, alongside the polarization trajectories on the Poincaré sphere. Unlike prior examples, the polarization states in this metasurface traverse a closed-looped trajectory connecting the north (0, 0, 1) and south (0, 0, −1) poles of the Poincaré sphere, indicating full control over the helicity of the field. These results validate the versatility of the designed 3D vectorial holographic projection in engineering complex polarization behavior.

It is worth noting that the longitudinal polarization response of each beam can be independently customized. Furthermore, the axial modulation rate can be further enhanced by incorporating a greater number of Bessel beam components in the superposition (as governed by Eq. (3)) and by reducing the structural period of the metasurface nanostructures (see Supplementary Section 1 for details). These enhancements enable faster polarization switching and higher axial resolution, offering substantial potential for advanced applications in vector beam shaping, volumetric data encoding, and 3D display technologies.

### All-optical information encryption

**Fig. 5:**
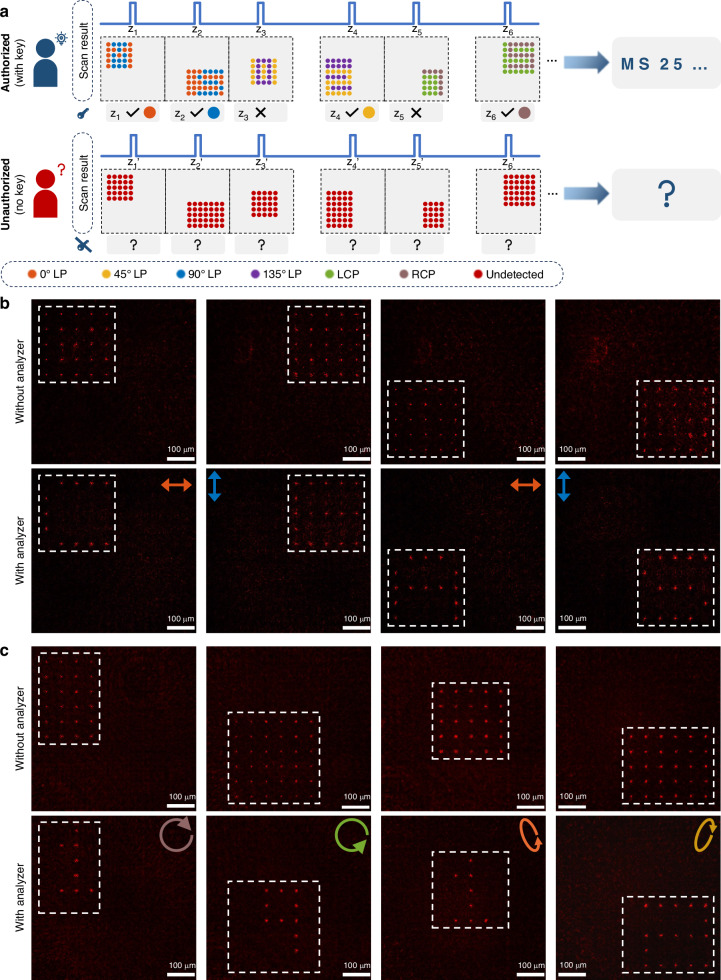
**Proof-of-concept demonstration of the polarization-gated optical encryption using metasurface-generated 3D vectorial light field.** **a** Schematic of a key-based optical encryption strategy, where axial intensity and polarization states jointly define the encoded ciphertext. Each symbol is accessible only with the correct decryption key (i.e., polarization filter and axial position). **b**, **c** Decoding results of (**b**) “CEAS” and (**c**) “1912” with and without the key. Scale bars: 100 μm. Without the correct key, the patterns appear randomized; correct polarization selection reveals hidden content with high contrast

Since the projected 3D vectorial patterns are fully determined by the axial intensity and polarization response functions of the programmable beam array, the proposed framework naturally supports advanced functionalities such as all-optical encryption and secure information storage. By increasing the size and complexity of the beam array, information can be encoded in a high-capacity, parallel, and secure manner. Figure 5a schematically illustrates a key-based holographic encryption strategy that leverages the longitudinal intensity and polarization degrees of freedom of the beam array. In this scheme, the metasurface is designed to project customized light-field patterns—directed by ciphertext content—at predefined axial depths, with each pattern associated with a distinct polarization state. To further conceal the encoded information, deceptive patterns are introduced with orthogonal polarization states or placed at misleading depths, thereby generating deliberate optical interference that obscures the true signal. Successful decryption requires a key specifying both the exact axial positions and polarization states of the symbols. Without this key, even direct physical access to the metasurface does not allow recovery of the ciphertext, ensuring hardware-level optical security.

As a proof of concept, we designed and fabricated two metasurfaces, each encoding different ciphertexts within a 10 × 10 beam array. The expanded array size significantly enhances encryption capacity through increased spatial and polarization multiplexing. In the first demonstration, the metasurface sequentially reconstructs the letters “C,” “E,” “A,” and “S” at the four corners of the array along the propagation axis, each encoded in a linear polarization state. In the second demonstration, the metasurface encodes the digits “1,” “9,” “1,” and “2” at more irregular positions with varying polarization states. To camouflage the content, surrounding beams are encoded with orthogonal polarizations to generate visually noisy patterns, ensuring that the overall transverse intensity distribution appears irregular and uninformative to direct observation or conventional imaging. Correct decoding requires knowledge of both the symbol depths and their corresponding polarization states.

Figure 5b, c shows the experimentally measured transverse intensity distributions at selected axial positions, with and without polarization filtering. Without the analyzer, the projected patterns display uniformly distributed intensity due to the intentional inclusion of polarization-mismatched decoy beams. When the correct analyzer is applied at the target axial planes, the decoy components are suppressed, and the true polarization channels are selectively transmitted, revealing the hidden symbols with high contrast. This polarization-gated decoding mechanism substantially improves encryption fidelity by enforcing physical-layer security constraints based on polarization and depth matching. Encryption robustness can be further enhanced by introducing randomized polarization distributions or embedding higher-dimensional polarization trajectories. Beyond encryption, the scalability of the beam array architecture makes it suitable for high-throughput optical data encoding. With continued advances in metasurface nanofabrication^49–52^, this strategy could be extended to larger-scale implementations, opening promising opportunities for secure communication, multi-channel optical storage, and optical steganography.

## Discussion

In summary, we propose and experimentally demonstrate a metasurface platform for 3D vectorial holography, enabling volumetric light-field reconstruction with simultaneous control over spatial intensity and polarization. The target holograms are discretized into beam arrays, wherein the axial intensity and polarization profiles of individual beams are independently tailored through programmable longitudinal response functions. Experimental validations confirm the platform’s capability to concurrently sculpt spatial intensity and polarization distributions, with robust broadband performance and high fidelity across multiple axial depths and spectral ranges. Furthermore, by integrating Jones matrix engineering with dual-matrix holography, we realize precise control over polarization trajectories, including linear, elliptical, and circular evolutions, within a compact, passive device.

Beyond conventional holographic reconstruction, we introduce a 3D vectorial encryption scheme that exploits polarization- and depth-encoded beam arrays for secure, key-based optical information encoding and retrieval. This strategy enables spatially multiplexed data storage with hardware-level security, where both polarization and axial information are required for successful decryption. With advances in nanofabrication, we envision that the proposed longitudinally customized 3D vectorial holographic projection will open new avenues for secure optical communication, high-density data storage, optical steganography, and quantum information processing.

## Methods

### Sample fabrication

The metasurface is fabricated using a multi-step process involving plasma-enhanced chemical vapor deposition (PECVD), electron beam lithography (EBL), electron beam evaporation (EBE), and inductively coupled plasma reactive ion etching (ICP-RIE). Beginning with a 500-μm-thick double-polished fused silica substrate, a layer of 400-nm-thick *α*-Si is deposited onto the substrate using a PECVD system. After oxygen plasma cleaning, a layer of hexamethyldisilazane is vapor-coated on the *α*-Si film to improve adhesion. A layer of 200-nm-thick positive E-beam resist and a thin layer of E-spacer are then spin-coated on the sample in turn. Next, the designed nanopattern is defined in the resist by implementing EBL and development. The 30-nm-thick aluminum as a hard mask with the reversed pattern is obtained via an EBE system combined with a lift-off process in n-methyl-pyrrolidone. ICP-RIE is used to pattern into the device layer using an optimized recipe. The chamber pressure is 10 mTorr, the flow ratio of C_4_F_8_/SF_6_ is 1.1, and the RF power of the ICP generator and bias is 2000 W and 100 W, respectively. Finally, the metasurface is obtained after removing the residual mask with the aluminum etchant.

## Supplementary information

Supplementary Materials for Longitudinally engineered metasurfaces for 3D vectorial holography

## Data Availability

All the data in this study are provided within the paper and its supplementary information. The data of this study are available from the corresponding author upon request.
