# Supplementary material for: Longitudinally engineered metasurfaces for 3D vectorial holography
Source: Light Sci Appl. 2026 Jan 3;15:36. doi: 10.1038/s41377-025-02158-5 (PMC12764939; doi:10.1038/s41377-025-02158-5)
Supplement: Supplementary file 1 — Supplementary Materials for Longitudinally engineered metasurfaces for 3D vectorial holography [file 41377_2025_2158_MOESM1_ESM.pdf]

Supplementary Information for

Longitudinally engineered metasurfaces for 3D vectorial holography

Table of Contents

Section 1: Longitudinal modulation theory for the tailored beam arrays

Section 2: Beam Dimensions and Design Considerations

Section 3: Construction of the matrix metasurface profile for longitudinal modulation

Section 4: Dual matrix holography of the compound phase metasurface

Section 5: Design of the unit cell in the metasurface

Section 6: Optical characterization of fabricated metasurfaces

Section 7: Characterization of independent longitudinal intensity modulation

Section 8: Full Stokes polarimetry along the *z*-axis

Section 9: Experimental conditions for projected 3D vectorial holography

Section 1: Longitudinal modulation theory for the tailored beam arrays

The longitudinal modulation theory is founded on the principle of spatial harmonic beating [1], which results from interference between two co-propagating plane waves of identical frequency and polarization but slightly different axial wavenumbers, k_z. This interference produces a modulated intensity envelope along the optical axis. By generalizing this principle, one can synthesize an arbitrary complex-valued axial profile via the superposition of harmonics equally spaced in the k_z-space [2-3], with spacing inversely proportional to the desired modulation range, Z_0.

To implement this approach, Bessel beams are selected as the basis functions due to their intrinsic diffraction-resistance over extended propagation distances. The longitudinally modulated field after the metasurface can be modeled as a coherent sum of Bessel beams

$$E(\rho,\varphi,z,t) = e^{-i\omega t}\sum_{m=-N}^{N} A_m J_l\left(k_\rho^m \rho\right) e^{il\varphi} e^{ik_z^m z} \tag{S1}$$

where A_m refers to the complex-valued scalar coefficient for each beam, $J_l(\cdot)$ refers to the lth-order Bessel function of the first kind in the series, and k_ρ^m and k_z^m are the transverse and longitudinal wavenumbers, satisfying the dispersion relation $\left(k_\rho^m\right)^2 + (k_z^m)^2 = \left(\frac{\omega}{c}\right)^2$. It is noted that the value of the topological charge l can be any integer to construct a beam with longitudinally tunable orbital angular momentum (OAM). In the paper, we set $l = 0$ for clarity in visualizing the transverse intensity patterns. The longitudinal modulation function $F(z)$ defines the target complex value intensity envelope. The coefficients A_m are obtained by projecting the target axial envelope $F(z)$ onto the exponential basis, yielding a Fourier-like synthesis as

$$A_m = \frac{1}{Z_0}\int_0^{Z_0} F(z) e^{-i\frac{2\pi}{Z_0}mz}\, \mathrm{d}z \tag{S2}$$

By substituting Equation (S2) into Equation (S1), the superposition at the central point of the field ($\rho = 0$) becomes a Fourier series representation of the envelope $F(z)$ for $l = 0$. Then, the complex amplitude profile of the metasurface can be derived by setting $z = 0$ in the Equation (S1). The output Bessel beams will constructively or destructively interfere along the propagation direction, directed by the longitudinal response function.

Also, by introducing the lateral displacements indexed by beam coordinates (p, q), we can create multiple parallel beams with independently programmable longitudinal responses. The field distribution of each displaced beam can be expressed as

$$E_{p,q}(\boldsymbol{\rho}, z) = \sum_{m=-N}^{N} A_{p,q,m} J_0\left(k_\rho^m \left\|\boldsymbol{\rho} - \boldsymbol{\rho}_{p,q}\right\|\right) e^{ik_z^m z} \tag{S3}$$

where $J_0(\cdot)$ refers to the zeroth-order Bessel function of the first kind, $\boldsymbol{\rho}_{p,q}$ refers to the lateral

displacement of the beam, and p and q refer to the row and column number of the beam in the array. Hence, we can obtain the expression of the field of the output structured beams array as the sum over all displaced beams

$$E(\boldsymbol{\rho}, z) = \sum_{p,q} E_{p,q}(\boldsymbol{\rho}, z)$$
$$= \sum_{p,q} \sum_{m=-N}^{N} A_{p,q,m} J_0\left(k_\rho^m \left\|\boldsymbol{\rho} - \boldsymbol{\rho}_{p,q}\right\|\right) e^{ik_z^m z} \quad \text{(S4)}$$

Then, the transmission profile of the corresponding metasurface can be computed by setting $z = 0$ in the above equation as $U_{\text{meta}}(\rho, \varphi) = E(\rho, \varphi, z = 0)$.

Since the longitudinal response function can be tailored continuously along the propagation direction, the metasurface can project holograms with arbitrary axial intensity and polarization profiles within a volumetric region, which means that the proposed design framework can readily be extended to continuous longitudinal modulation. To illustrate this capability, we performed simulations demonstrating a rotating 3D helical hologram generated by continuous modulation. As illustrated in Fig. S1, the 3D hologram corresponding to a helix with varying angles were projected sequentially along the propagation direction by continuously modifying the response function of each beam.

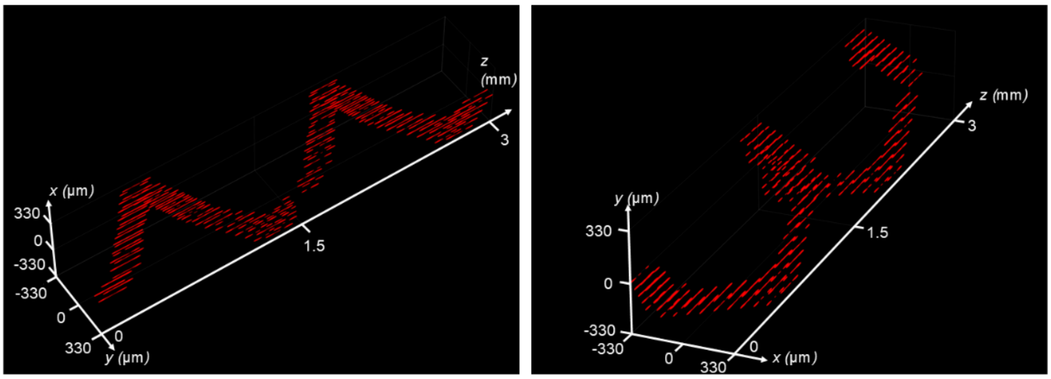

Fig. S1 | Simulated demonstration of continuous 3D holographic projection. The volume-rendered images show a rotating 3D helix reconstructed by the metasurface from different viewing angles.

Section 2: Beam Dimensions and Design Considerations

The maximum range of the generated by the metasurface to realize the modulation is defined by Z_0. It is noted that the required aperture size of the metasurface is bounded from below by

$$D \geq 2Z_0\sqrt{\left(\frac{k_0}{k_z^{m=-N}}\right)^2 - 1} \tag{S5}$$

where $k_z^{m=-N}$ is the smallest longitudinal wavenumber. The above equation sets the lower limit on the aperture size of the metasurface which guarantees the generation of the output beam over the range Z_0. As described in Equation (S5), the maximum longitudinal modulation range depends on the metasurface size, operational wavelength, and lattice constant limit. This relationship is illustrated in Fig. S2a. To validate it, we conducted additional simulations examining how the maximum longitudinal modulation range varies with metasurface size, as shown in Fig. S2b.

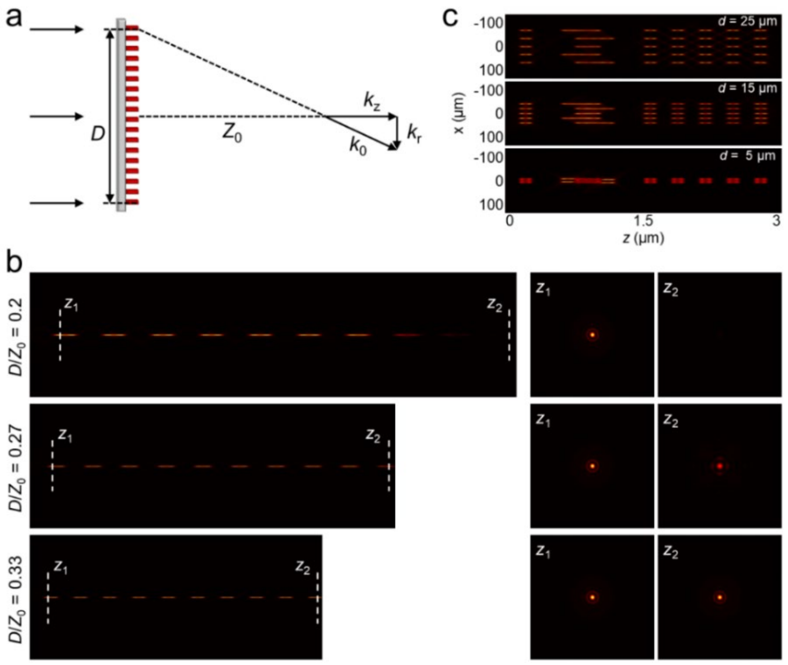

Fig. S2 | Simulated longitudinally modulated beams with varying maximum ranges and lateral spacing. a Schematic of the longitudinally modulated non-diffracting beams (Bessel modes), which can be generated by axicons with different cone angles. **b** Simulated longitudinal and transverse intensity profiles after tuning the longitudinal beam range. **c** Simulated longitudinal intensity profiles of the modulated beam array for different lateral spacings.

Furthermore, the relation between the center wavevector $k_z^{m=0}$ and the beam's spot size ρ_0 can be expressed as

$$k_z^{m=0} = \sqrt{(k_0)^2 - \left(\frac{2.4}{\rho_0}\right)^2} \tag{S6}$$

where the value 2.4 approximates the first zero crossing of the zeroth order Bessel function. The beam's spot size ρ_0 also determines the spacing between adjacent beams in the projected array. In practice, the lateral spacing is increased during longitudinal polarization modulation to ensure the independence of each beam modulation and to minimize mutual interference. The simulation results, shown in Fig. S2c, illustrate the interference behavior between adjacent beams under different spacing conditions.

Regarding polarization resolution, the metasurface exploits spatial beating among co-propagating Bessel beams with different k_z values. To resolve high spatial frequency polarization patterns, the square lattice constant P of nanopillars on the metasurface is set to 250 nm. Therefore, the Bessel functions with large spatial frequency, up to $k_r \leq \frac{2\pi}{2\times 250\ \mathrm{nm}}$, can be supported without violating Nyquist's sampling criterion. However, due to the checkerboard sampling pattern in the dual matrix holography, the maximum value of k_r needs be to further reduced by another factor of 2, yielding a maximum spatial frequency of $k_r \approx 6\times10^6$ rad·m^{-1}. This allowed the metasurface to control polarization with a rotation frequency of 0.36°·μm^{-1} in the paper. With additional demagnification, even finer axial control can be achieved, scaling quadratically with the demagnification factor.

Design Considerations. The main design limitations stem from the lateral spacing between beams and the superposition parameter (N). Increasing the lateral spacing reduces unwanted interference but sacrifices image sampling density, leaving large black background and a few beams, whereas reducing it enhances spatial sampling but introduces stronger crosstalk.

Several directions can be pursued to address this challenge. Firstly, introducing cross-polarization between adjacent beams can help suppress interference while reducing the lateral spacing. Secondly, advanced fabrication methods can be employed to realize larger metasurfaces, thereby relaxing the lateral-spacing constraint. Thirdly, suitable optical configurations can be introduced to project the desired hologram onto a larger or smaller volume to help improve performance.

A larger superposition parameter N increases the accessible frequency range and reconstruction quality but demands smaller lattice constants and larger metasurface sizes. High-resolution holography can be achieved by setting a shorter longitudinal modulation range (Z_0) and using dense beam arrays capable of supporting higher spatial frequencies. Furthermore, advances in nanofabrication enabling smaller feature sizes will further mitigate these constraints.

Section 3: Construction of the matrix metasurface profile for longitudinal modulation

Following the longitudinal modulation theory in Section 1, the axial behavior of each beam is defined by a longitudinal response function $F(z)$. This enables the generation of structured beams with an arbitrary complex value profile along the propagation axis. A scalar function is employed to $F_{p,q}(z)$ quantitatively define the longitudinal intensity response of the (p, q)-th beam, while a 1×2 Jones vectorial function $\vec{J}_{p,q}(z)$ is employed to describe the spatially varying polarization state along the propagation axis. Therefore, the complete longitudinal response function of the (p, q)-th beam can be obtained by combining the two functions as $\vec{F}(z) = F_{p,q}(z)\vec{J}_{p,q}(z)$.

Since the polarization state on the Poincaré sphere can be represented as a linear coherent superposition of two orthogonal circular polarization basis states, the z-denpendent complex weighting coefficients $a^{\mathrm{L}}_{p,q}(z)$ and $a^{\mathrm{R}}_{p,q}(z)$ of the left- and right-circular polarization (LCP and RCP) state can be calculated based on $\vec{J}(z)$ as follows

$$\vec{J}(z) = a^{\mathrm{L}}_{p,q}(z)|L\rangle + a^{\mathrm{R}}_{p,q}(z)|R\rangle \tag{S7}$$

where $\left|L\right\rangle = \frac{1}{\sqrt{2}}\begin{bmatrix}1\\ i\end{bmatrix}$ and $\left|R\right\rangle = \frac{1}{\sqrt{2}}\begin{bmatrix}1\\ -i\end{bmatrix}$ denote the unit vectors for LCP and RCP in the linear polarization basis, respectively. The weighting coefficients satisfy the normalization condition $|a^{L}_{p,q}(z)|^2 + |a^{R}_{p,q}(z)|^2 = 1$. Then, the longitudinal response function $\vec{F}(z)$ can be decomposed into two complex value response functions in two orthogonal LCP and RCP channels as

$$\vec{F}_{p,q}(z) = F_{p,q}(z)\vec{J}(z) = F_{p,q}(z)\left[a^{\mathrm{L}}_{p,q}(z)|L\rangle + a^{\mathrm{R}}_{p,q}(z)|R\rangle\right] = F^{\mathrm{L}}_{p,q}(z)|L\rangle + F^{\mathrm{R}}_{p,q}(z)|R\rangle \tag{S8}$$

where $F^{\mathrm{L}}_{p,q}(z) = F_{p,q}(z)a^{\mathrm{L}}_{p,q}(z)$ and $F^{\mathrm{R}}_{p,q}(z) = F_{p,q}(z)a^{\mathrm{R}}_{p,q}$.

As illustrated in Section 1, the complex weighting coefficients of these Bessel components are determined by projecting the desired longitudinal response functions onto the basic exponential functions as

$$A^{\mathrm{L/R}}_{p,q,m} = \frac{1}{Z_0}\int_0^{Z_0} F^{\mathrm{L/R}}_{p,q}(z)e^{-i\frac{2\pi}{Z_0}mz}\mathrm{d}z \tag{S9}$$

where $A^{\mathrm{L/R}}_{p,q,m}$ are complex-valued coefficients that control the amplitude and phase of the m-th Bessel beam component in the LCP or RCP channels. Hence, the complete generated 3D vectorial light field can be expressed as the coherent summation of all the spin-dependent Bessel contributions across the full beam array, which can be written as

$$\vec{E}(\boldsymbol{\rho}, z) = \sum_{p,q}\sum_{m=-N}^{N}\left[A^{\mathrm{L}}_{p,q,m}J_0\left(k^m_\rho\left\|\boldsymbol{\rho} - \boldsymbol{\rho}_{p,q}\right\|\right)e^{ik^m_z z}|L\rangle + A^{\mathrm{R}}_{p,q,m}J_0\left(k^m_\rho\left\|\boldsymbol{\rho} - \boldsymbol{\rho}_{p,q}\right\|\right)e^{ik^m_z z}|R\rangle\right]$$

$$= \sum_{p,q} \sum_{m=-N}^{N} J_0\left(k_\rho^m \left\|\boldsymbol{\rho} - \boldsymbol{\rho}_{p,q}\right\|\right) e^{ik_z^m z} \cdot \left[A_{p,q,m}^{\mathrm{L}}|L\rangle + A_{p,q,m}^{\mathrm{R}}|R\rangle\right] \tag{S10}$$

Then, the transmission profile of the metasurface in the LCP or RCP channels can be similarly obtained by substituting $z = 0$ into Equation (S10) as

$$U_{\mathrm{meta}}^{\mathrm{L}}(\boldsymbol{\rho}) = \sum_{p,q} \sum_{m=-N}^{N} A_{p,q,m}^{\mathrm{L}} J_0\left(k_\rho^m \left\|\boldsymbol{\rho} - \boldsymbol{\rho}_{p,q}\right\|\right) \tag{S11a}$$

$$U_{\mathrm{meta}}^{\mathrm{R}}(\boldsymbol{\rho}) = \sum_{p,q} \sum_{m=-N}^{N} A_{p,q,m}^{\mathrm{R}} J_0\left(k_\rho^m \left\|\boldsymbol{\rho} - \boldsymbol{\rho}_{p,q}\right\|\right) \tag{S11b}$$

Finally, the 2×2 complex Jones matrix profile of the metasurface $\tilde{J}_{\mathrm{meta}}(\rho, \varphi)$ can be determined based on the profiles in the two polarization channels and the required incident polarization state. Considering the case of x-polarized incident light for instance, by requiring the beam transformation as $\tilde{J}_{\mathrm{meta}}(\rho, \varphi)|L\rangle = U_{\mathrm{meta}}^{\mathrm{R}}(\rho, \varphi)|R\rangle$ and $\tilde{J}_{\mathrm{meta}}(\rho, \varphi)|R\rangle = U_{\mathrm{meta}}^{\mathrm{L}}(\rho, \varphi)|L\rangle$, the Jones matrix profile of the metasurface $\tilde{J}_{\mathrm{meta}}$ takes the form as follows [4-5]

$$\tilde{J}_{\mathrm{meta}} = \frac{1}{2}\begin{bmatrix} \left(U_{\mathrm{meta}}^{\mathrm{R}} + U_{\mathrm{meta}}^{\mathrm{L}}\right) & -i\left(U_{\mathrm{meta}}^{\mathrm{R}} - U_{\mathrm{meta}}^{\mathrm{L}}\right) \\ -i\left(U_{\mathrm{meta}}^{\mathrm{R}} - U_{\mathrm{meta}}^{\mathrm{L}}\right) & -\left(U_{\mathrm{meta}}^{\mathrm{R}} + U_{\mathrm{meta}}^{\mathrm{L}}\right) \end{bmatrix} \tag{S12}$$

The transmission matrix profile of the metasurface $\tilde{J}_{\mathrm{meta}}(\rho, \varphi)$ allows precise spatial modulation of both intensity and polarization along the propagation axis, enabling 3D holographic projection with full vectorial field manipulation.

As the proof-of-concept demonstration, we designed three metasurfaces to generate a beam array longitudinally evolving along different polarization trajectories on the Poincaré sphere. The target z-dependent azimuthal angle (ψ) and ellipticity (χ) profiles of the output structured beam array along the propagation direction are plotted in Fig. S3. Note that angles are only plotted within axial regions showing non-zero intensity.

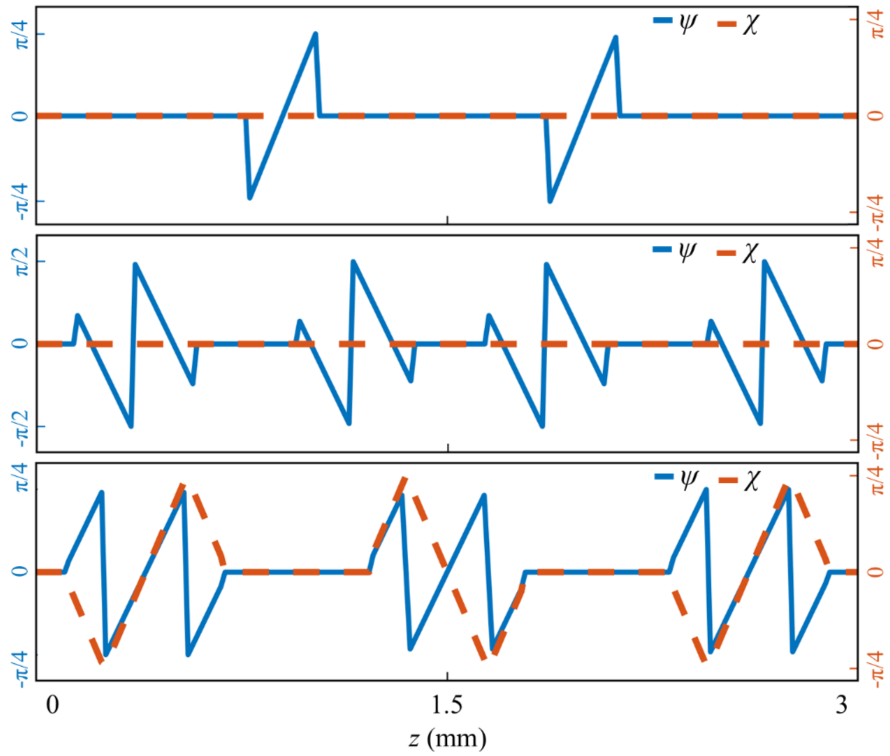

Fig. S3 | Target polarization evolution along the optical axis. The tailored azimuthal angle (ψ) and ellipticity (χ) profiles of the structured beam array output by the three metasurfaces along the propagation direction. a The target profiles of the first metasurface generating beam array with longitudinally varying patterns ("N" and "J") and rotating linear polarization states. **b** The target profiles of the second metasurface generating beam array with longitudinally varying patterns ("M," "E," "T," and "A") and rotating linear polarization states. **c** The target profiles of the third metasurface generating beam array with longitudinally varying patterns ("O," "P," and "T") and elliptical polarization states.

Section 4: Dual matrix holography of the compound phase metasurface

To achieve spatially varying polarization transformations using birefringent nanopillars with locally rotated fast axes, each element of the metasurface is described by a Jones matrix [6-7] as follows

$$\tilde{T}(\theta) = \tilde{R}(-\theta)\begin{bmatrix} t_x e^{i\phi_x} & 0 \\ 0 & t_y e^{i\phi_y} \end{bmatrix}\tilde{R}(\theta) \tag{S13}$$

where t_x and t_y refer to the transmission amplitudes of a single nanopillar for light polarized parallel and perpendicular to the fast axis, respectively. ϕ_x and ϕ_y refer to the phase retardance of the nanopillar for light polarized parallel and perpendicular to the fast axis. The rotation matrix $\tilde{R}(\theta) = \begin{bmatrix} \cos\theta & -\sin\theta \\ \sin\theta & \cos\theta \end{bmatrix}$ is determined by the local in-plane rotation angle θ of the nanopillar concerning the fast axis. The matrix $\tilde{T}$ is a unitary matrix and can be diagonalized by solving the characteristic equation. The eigenvalues and eigenvectors of this Jones matrix $\tilde{T}$ require that the elements of the metasurface spatial filter have a birefringent response with the phase shifts (ϕ_x, ϕ_y) along the two perpendicular symmetry axes and the rotation angle θ of fast axes as a function of the reference coordinate.

This formalism assumes unitary behavior, meaning that the matrix must preserve energy (i.e., no amplitude modulation). However, the ideal Jones matrix profiles obtained from longitudinal modulation theory may be non-unitary or complex-valued, making direct physical implementation challenging. To help realize arbitrary complex-valued Jones matrices in a passive metasurface, the dual matrix holography [8-10] provides a method that enables the decomposition of arbitrary 2×2 complex symmetric matrices into a sum of two unitary matrices. These unitary matrices, whose eigenvalues are pure phase terms, can then be directly mapped to metasurface geometries composed of birefringent waveplate-like unit cells.

Generally, through the singular value decomposition, the Jones matrices can be factorized in the form $\widetilde{\mathrm{M}} = \widetilde{\mathrm{W}}\tilde{\Sigma}\widetilde{\mathrm{V}}^{\dagger}$, where $\dagger$ denotes the conjugate transpose, and $\tilde{\Sigma}$ is a real diagonal matrix that has the nonnegative and real singular values of $\widetilde{\mathrm{M}}$. Furthermore, without loss of generality, it is assumed that the target matrix is normalized. In this case, a general normalized matrix $\widetilde{\mathrm{M}}$ can be factorized into the following form

$$\widetilde{\mathrm{M}} = \widetilde{\mathrm{W}}\tilde{\Sigma}\widetilde{\mathrm{V}}^{\dagger} = \widetilde{\mathrm{W}}\begin{bmatrix} r_1 & 0 \\ 0 & r_2 \end{bmatrix}\widetilde{\mathrm{V}}^{\dagger} \tag{S14}$$

where r_1 and r_2 are the normalized real singular values of $\widetilde{\mathrm{M}}$ (i.e., $0 \leq r_{1,2} \leq 1$). Any normalized real number can be expressed as a sum of two conjugate complex numbers whose magnitudes are the

same. Therefore, r_1 and r_2 can be expressed as

$$r_1 = R_1 + R_2 = \frac{r_1 + i\sqrt{1-r_1^2}}{2} + \frac{r_1 - i\sqrt{1-r_1^2}}{2} \tag{S15a}$$

$$r_2 = T_1 + T_2 = \frac{r_2 + i\sqrt{1-r_2^2}}{2} + \frac{r_2 - i\sqrt{1-r_2^2}}{2} \tag{S15b}$$

where the factor of 2 in the denominator is part of the normalization and is common to all terms. Hence, it can simply be factored out, and it does not imply any amplitude modulation. Therefore, $\widetilde{\mathrm{M}}$ can be expressed as

$$\widetilde{\mathrm{M}} = \widetilde{\mathrm{W}} \begin{bmatrix} R_1 & 0 \\ 0 & T_1 \end{bmatrix} \widetilde{\mathrm{V}}^\dagger + \widetilde{\mathrm{W}} \begin{bmatrix} R_2 & 0 \\ 0 & T_2 \end{bmatrix} \widetilde{\mathrm{V}}^\dagger \tag{S16}$$

Here, $R_{1,2}$ and $T_{1,2}$ are phase-only quantities, and $\widetilde{\mathrm{W}}$ and $\widetilde{\mathrm{V}}^\dagger$ are unitary matrices that only introduce a rotation of bases. Therefore, a general complex matrix $\widetilde{\mathrm{M}}$ is now expressed as a sum of two unitary matrices. Finally, obeying Nyquist's sampling criterion in constructing the interlaced Jones matrices on the metasurface permits the complex Fourier spectrum of the original Jones matrix distribution to be fully reconstructed in the far field. Therefore, the longitudinally modulated field can be retrieved in real space to be measured by the CCD.

The simplified procedure of the dual matrix holography to transform the target distribution of spatially varying 2×2 complex Jones matrices into a unitary-only distribution is summarized as follows

(1) The desired matrix distribution $\tilde{J}_{\mathrm{meta}}(\rho, \varphi, z = 0)$ is obtained by evaluating Equation (S12).

(2) $\tilde{J}_{\mathrm{meta}}(\rho, \varphi, z = 0)$ is then normalized, locally, by dividing by the global maximum eigenvalue: $\tilde{J}_{\mathrm{norm}}(\rho, \varphi) = \frac{\tilde{J}_{\mathrm{meta}}(\rho, \varphi, z=0)}{\max\{\mathrm{eig}(\tilde{J}_{\mathrm{meta}})\}}$, $\forall \rho$. This normalization step is required as the passive metasurface only introduces a loss-like effect with no gain.

(3) At each location in the transverse plane, $\tilde{J}_{\mathrm{norm}}(\rho, \varphi)$ decomposes into two unitary matrices using singular value decomposition. This decomposition serves as a factorization for each 2×2 normalized Jones matrix such that $\tilde{J}_{\mathrm{norm}} = [\widetilde{\mathrm{W}}\widetilde{\mathrm{D}}\widetilde{\mathrm{V}}^\dagger]$, where $\widetilde{\mathrm{D}}$ is now a 2×2 diagonal matrix with non-negative real singular values.

(4) The singular values of $\tilde{u}$, D_{11} and D_{22}, at each location, decompose into a sum of two complex values $C_{nn}^{(1)} = \frac{D_{nn} + i\sqrt{1-D_{nn}^2}}{2}$ and $C_{nn}^{(2)} = \frac{D_{nn} - i\sqrt{1-D_{nn}^2}}{2}$, where *n* denotes the index of the diagonal entry (*nn* = 11 or 22). Here, the spatial dependence on ρ has been omitted for clarity.

(5) The complex values $C_{nn}^{(1,2)}$ then become the new diagonal entries of two unitary matrices, with the same rotation matrices $\widetilde{\mathrm{W}}$ and $\widetilde{\mathrm{V}}^\dagger$, such that

$$\tilde{J}_{\text{norm}}(\rho,\varphi)=\tilde{u}_1+\tilde{u}_2=\widetilde{\mathrm{W}}\begin{bmatrix} C_{11}^{(1)}(\rho,\varphi) & 0 \\ 0 & C_{22}^{(1)}(\rho,\varphi)\end{bmatrix}\widetilde{\mathrm{V}}^{\dagger}+\widetilde{\mathrm{W}}\begin{bmatrix} C_{11}^{(2)}(\rho,\varphi) & 0 \\ 0 & C_{22}^{(2)}(\rho,\varphi)\end{bmatrix}\widetilde{\mathrm{V}}^{\dagger} \quad \text{(S17)}$$

By construction, $D_{nn}=C_{nn}^{(1)}+C_{nn}^{(2)}$ and $\left|C_{nn}^{(1,2)}=1\right|$, $\forall\rho$. As such, $\tilde{u}(\rho,\varphi)$ decomposes into two unitary matrices, $\tilde{u}_1$ and $\tilde{u}_2$, where each can be implemented using our proposed dielectric metasurface comprised of wave-plate-like unit cells. Through judicious interlacing of $\tilde{u}_1$ and $\tilde{u}_2$ on the same metasurface, the intended polarization behavior of the device can be achieved at the far field. To achieve this, $\tilde{u}_1$ and $\tilde{u}_2$ were periodically sampled employing two complementary draughts board patterns, M_1 and M_2, expressed as

$$M_{1,2}(\rho,\varphi)=\frac{1}{2}\sum_{m=-\infty}^{\infty}\sum_{l=-\infty}^{\infty}\mathbb{I}\left[\Lambda_{1,2}(m,l)\right]\mathrm{e}^{i\frac{2\pi\rho}{p}(m\cos\varphi+l\sin\varphi)} \quad \text{(S18)}$$

$$\Lambda_{1,2}(m,l)=\cos\left[\frac{\pi(m\pm l)}{2}\right]\operatorname{sinc}\left(\frac{m\pi}{2}\right)\operatorname{sinc}\left(\frac{l\pi}{2}\right) \quad \text{(S19)}$$

where $\mathbb{I}$ is the 2×2 identity matrix, and p defines the periodicity of the draughts board pattern. In essence, Equation (S17) renders two complementary draughts board patterns whose transverse profile at each location alternates between $\mathbb{I}$ and the zero matrix. Multiplying $\tilde{u}_{1,2}$ by $M_{1,2}$, locally, and adding becomes equivalent to interlacing $\tilde{u}_1$ and $\tilde{u}_2$ onto the same surface with a period of p. Notably, allowing p to fulfill the Nyquist limit enables the full reconstruction of the transfer function of $\tilde{u}(\rho,\varphi)$ in the far field. The interlaced pattern at the metasurface plane is given by

$$\widetilde{U}_{\text{meta}}=M_1(\rho,\varphi)\tilde{u}_1(\rho,\varphi)+M_2(\rho,\varphi)\tilde{u}_2(\rho,\varphi) \quad \text{(S20)}$$

By taking the Fourier transform of the matrix $\widetilde{U}_{\text{meta}}$, it can be shown that the desired spectrum can be fully reconstructed provided that p satisfies the Nyquist criteria. The original matrix $\tilde{J}_{\text{meta}}(\rho,\varphi,z=0)$ can then be retrieved by performing an inverse Fourier operation.

Section 5: Design of the unit cell in the metasurface

The metasurfaces are composed of rectangular nanopillars fabricated from amorphous silicon (α-Si) nanopillars on the fused-silicon substrate. To inform the design, the complex refractive index curves of the α-Si film are first measured via spectroscopic ellipsometry, as shown in Fig. S4.

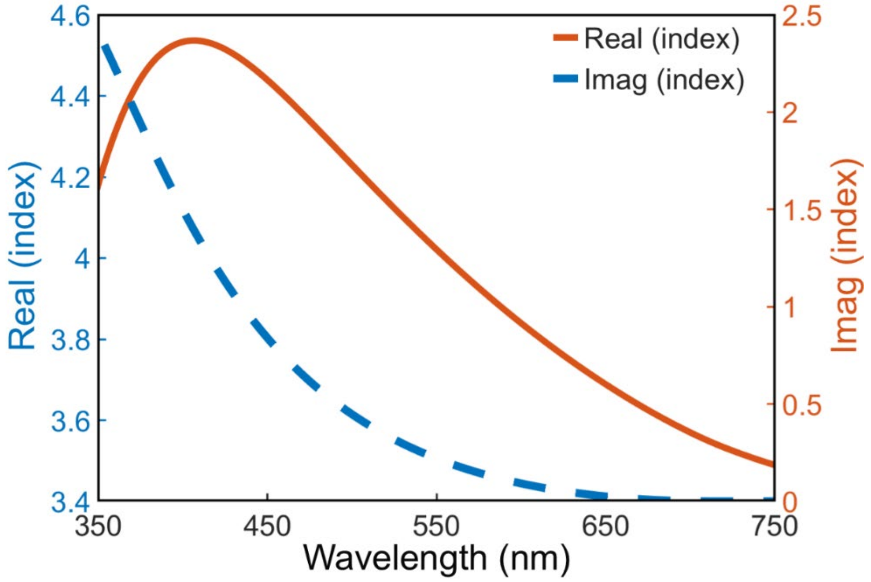

Fig. S4 | Complex refractive index of α-Si. The real (blue line) and imaginary (red line) parts of the complex refractive index as a function of wavelength are measured by spectroscopic ellipsometry.

The optical response of each nanopillar is tailored by its geometry, allowing independent phase modulation of orthogonal polarization components. The α-Si nanopillars on the substrate are arranged in a square lattice with a fixed period of P =250 nm and a fixed height of H = 400 nm. These parameters were selected to ensure subwavelength operation and satisfy the Nyquist criterion for spatial sampling of high-frequency polarization components. The rest of the structure parameters of the α-Si nanopillars are determined according to the target phase shift values. We utilized the finite-difference time-domain (FDTD) method to find the suitable parameters providing the target phase response for both x- and y-polarized input light. The nanopillar's width and length are both swept from 50 nm to 200 nm with a step of 5 nm to probe the desired phase shift. Periodic boundary conditions were applied in the lateral directions, while a perfectly matched layer was used along the vertical direction. Each unit cell was illuminated sequentially by x- and y-polarized plane waves to extract the polarization-dependent transmission phase and amplitude, and the corresponding simulated results are shown in Fig. S5.

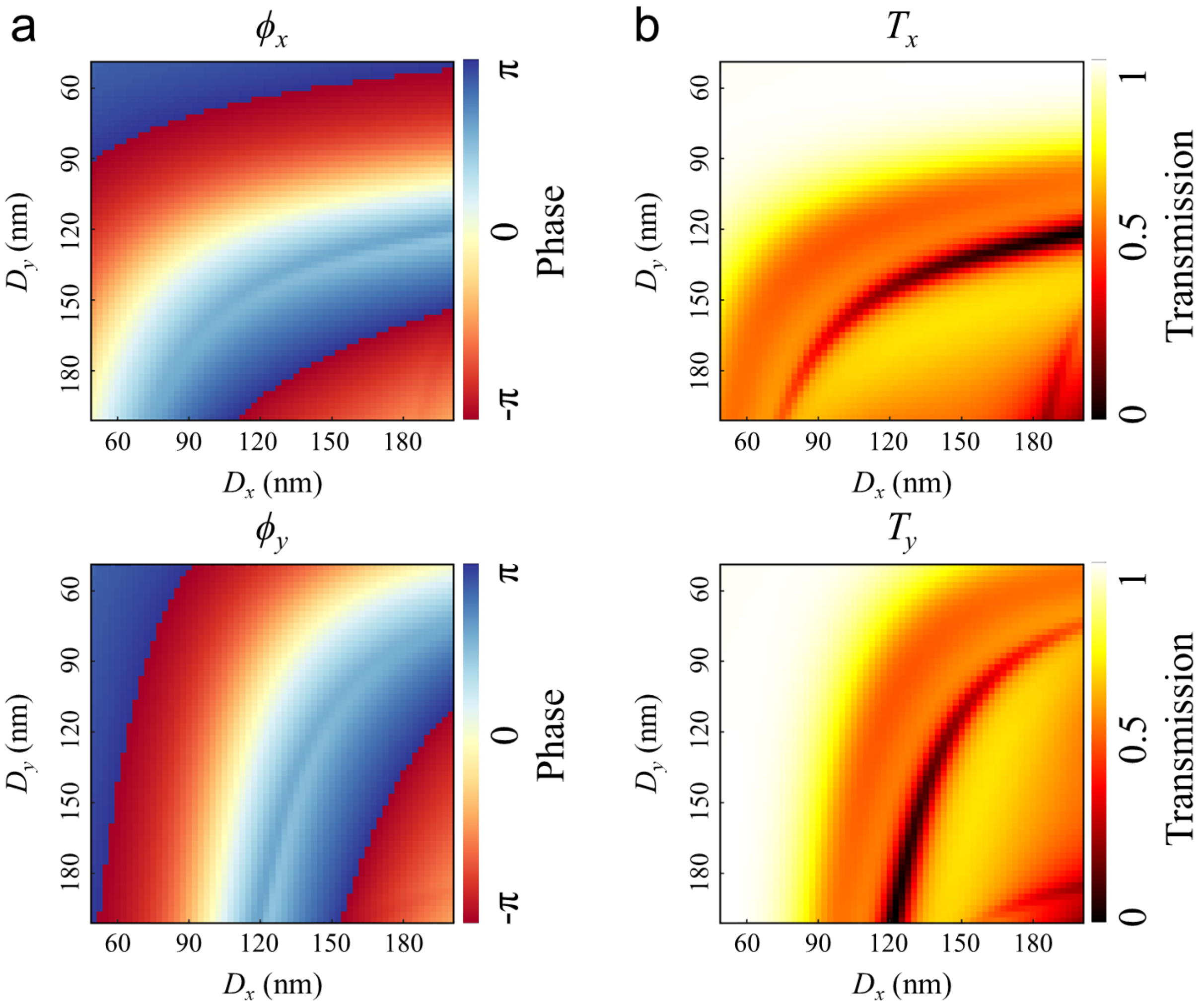

Fig. S5 | a Phase shift and **b** power transmission coefficient for *x*-polarized (top panel) and *y*-polarized light (bottom panel) as functions of the rectangular nanopillar parameters (D_x and D_y) at the wavelength of 633 nm.

To discretize the continuous phase response for practical implementation, the transmission phase was quantized into 8 levels, ranging from 0 to 2π with a π/4 step size. A lookup table (Table S1) is then constructed to map the desired phase shifts (ϕ_x, ϕ_y) to corresponding nanopillar geometries (D_x, D_y). This discrete phase coverage enables independent modulation of each polarization component across the metasurface aperture.

Table. S1 | Nanopillar geometries corresponding to discrete phase shifts in both *x*- and *y*- channels.

ϕ_y \ ϕ_x	π/4	π/2	3π/4	π	5π/4	3π/2	7π/4	2π
π/4	(125, 125)	(135, 120)	(175, 110)	(200, 105)	(80, 155)	(95, 140)	(105, 130)	(115, 125)
π/2	(120, 135)	(140, 140)	(165, 120)	(195, 115)	(75, 185)	(90, 160)	(100, 150)	(110, 140)
3π/4	(110, 175)	(120, 165)	(150, 150)	(175, 145)	(200, 140)	(85, 200)	(95, 185)	(105, 175)
π	(105, 200)	(115, 195)	(145, 175)	(165, 165)	(195, 155)	(130, 65)	(150, 60)	(165, 60)
5π/4	(155, 80)	(185, 75)	(140, 200)	(155, 195)	(95, 95)	(110, 90)	(125, 85)	(135, 85)
3π/2	(140, 95)	(160, 90)	(200, 85)	(65, 130)	(90, 110)	(105, 105)	(115, 100)	(125, 100)
7π/4	(130, 105)	(150, 100)	(185, 95)	(60, 150)	(85, 125)	(100, 115)	(110, 110)	(120, 110)
2π	(125, 115)	(140, 110)	(175, 105)	(60, 165)	(85, 125)	(100, 125)	(110, 120)	(115, 115)

Furthermore, we obtain the simulated normalized energy distribution in the pixel with one of the selected nanopillars with lateral dimensions $(D_x,\ D_y)$ = (140 nm, 95 nm), which corresponds to the propagation phase pair $(\phi_x,\ \phi_y)$ = (π/4, 3π/2). Figure S6 shows that the normalized magnetic energy density is mainly confined inside the nanopillar. This simulation result is due to the high index contrast of α-Si compared to its surrounding medium (which is air in this case). Therefore, each nanopillar is considered a stand-alone truncated waveguide. Thus, the waveguide-like behavior and negligible coupling validate the treatment of nanopillars as independent optical elements during the design.

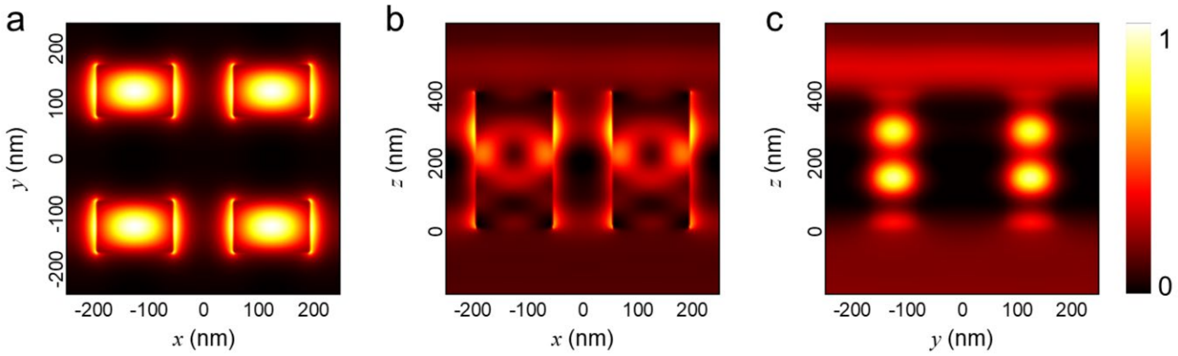

Fig. S6 | The normalized magnetic energy density distribution of the periodic arrays of the nanopillars for plane wave illumination at the wavelength of 633 nm. a *x*-*y* plane, **b** *x*-*z* plane, **c** *y*-*z* plane.

Section 6: Optical characterization of fabricated metasurfaces

To experimentally validate the proposed design framework, we fabricated six metasurfaces, each engineered to implement a distinct longitudinal modulation of intensity and polarization. These metasurfaces were patterned onto α-Si films using standard nanofabrication processes as described in the Methods section of the main text.

Photographs of the fabricated samples are presented in Fig. S7. Each metasurface spans approximately 1.2 mm×1.2 mm and is composed of a square lattice of rectangular nanopillars with 250 nm periodicity. These samples collectively demonstrate the versatility of the metasurface platform in producing structured light fields with programmable vectorial and scalar characteristics across the propagation axis.

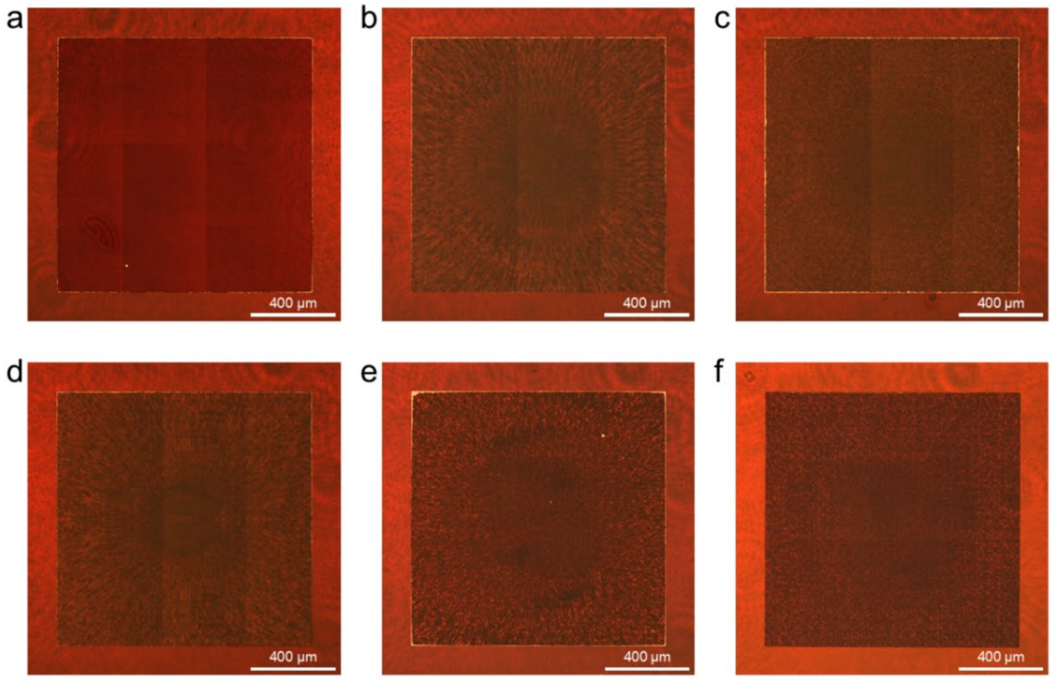

Fig. S7 | Optical photographs of the fabricated metasurfaces in the experiment. a The metasurface designed to generate a beam array with constant polarization and axially distributed patterns forming the word “OPTICS”. **b** The metasurface designed to generate a beam array with longitudinally varying patterns (“N” and “J”) and rotating linear polarization states. **c** The metasurface designed to generate a beam array with longitudinally varying patterns (“M,” “E,” “T,” and “A”) and rotating linear polarization states. **d** The metasurface designed to generate a beam array with longitudinally varying patterns (“O,” “P,” and “T”) and elliptical polarization states. **e** The metasurface generating beam array with encrypted messages “CEAS” in the longitudinally tunable intensity and polarization profiles. **f** The metasurface designed to generate a beam array with encrypted messages “1912” in the longitudinally tunable intensity and polarization profiles.

Section 7: Characterization of independent longitudinal intensity modulation

To evaluate beam-specific axial control, we conduct the *z*-axis scanning to record the intensity profiles of selected beams positioned at the beam coordinates (p = 0, q = 0), (p = 0, q = 2), and (p = -2, q = -2), respectively. The three beams are highlighted in Fig. S8a, Fig. S9a, and Fig. S10a with different colors, while the corresponding simulated and measured intensity profiles are plotted in Fig. S8b, Fig. S9b, and Fig. S10b. The experimentally measured intensity results confirm close agreement with target profiles along the propagation direction, verifying the independent longitudinal intensity modulation ability, which plays a central role in the formation of 3D holographic projections.

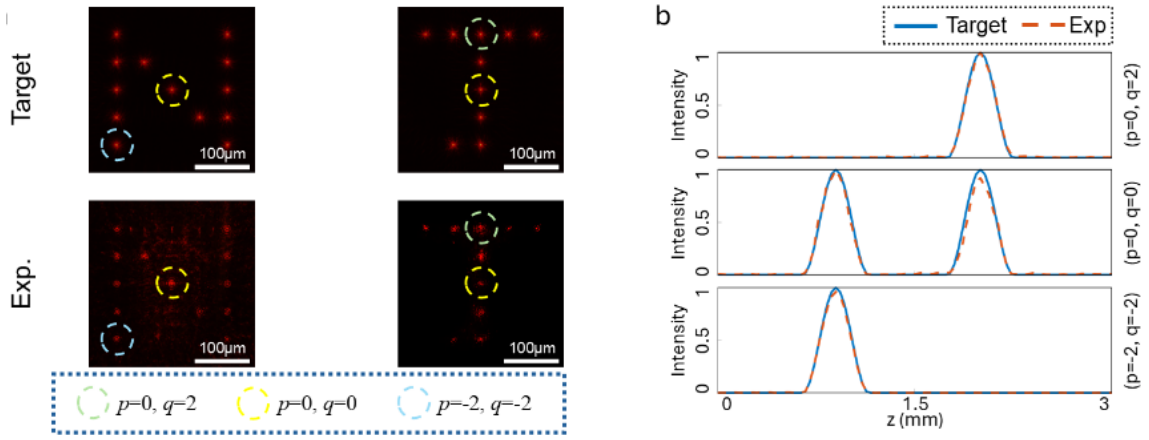

Fig. S8 | Measured and target intensity distribution of the array generated by the first metasurface, forming longitudinally varying patterns ("N" and "J"). a The target and experimentally measured transverse intensity distributions at the target axial locations. Three beams corresponding to the beam coordinates (p = 0, q = 0), (p = 0, q = 2), and (p = -2, q = -2) are highlighted by the yellow, green, blue dashed circles. The abbreviation "Exp" has the full name of "Experiment". **b** The experimentally measured normalized intensity profiles of the three beams, compared with the target intensity lines.

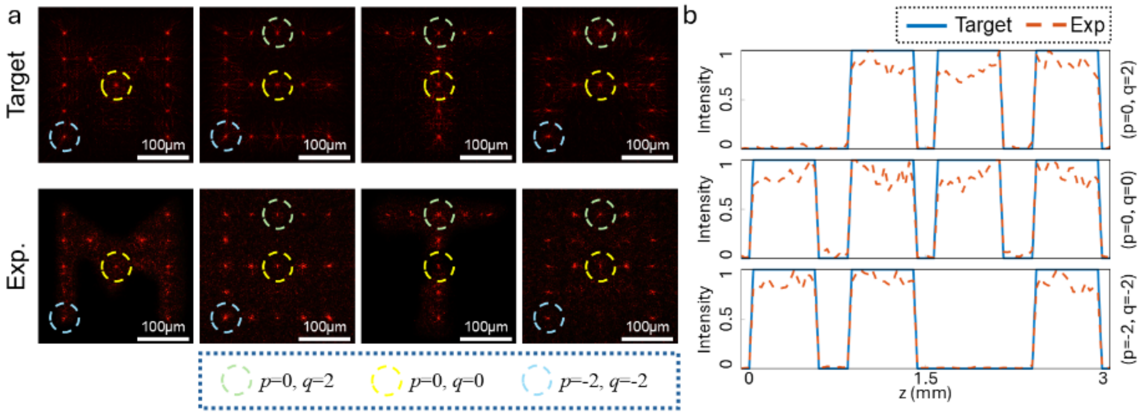

Fig. S9 | Measured and target intensity distribution of the array generated by the second metasurface, forming longitudinally varying patterns ("M," "E," "T," and "A"). a The target and experimentally measured transverse intensity distributions at the target axial locations. Three beams corresponding to the beam coordinates ($p = 0$, $q = 0$), ($p = 0$, $q = 2$), and ($p = -2$, $q = -2$) are highlighted by the yellow, green, blue dashed circles. **b** The experimentally measured normalized intensity profiles of the three beams, compared with the target intensity lines.

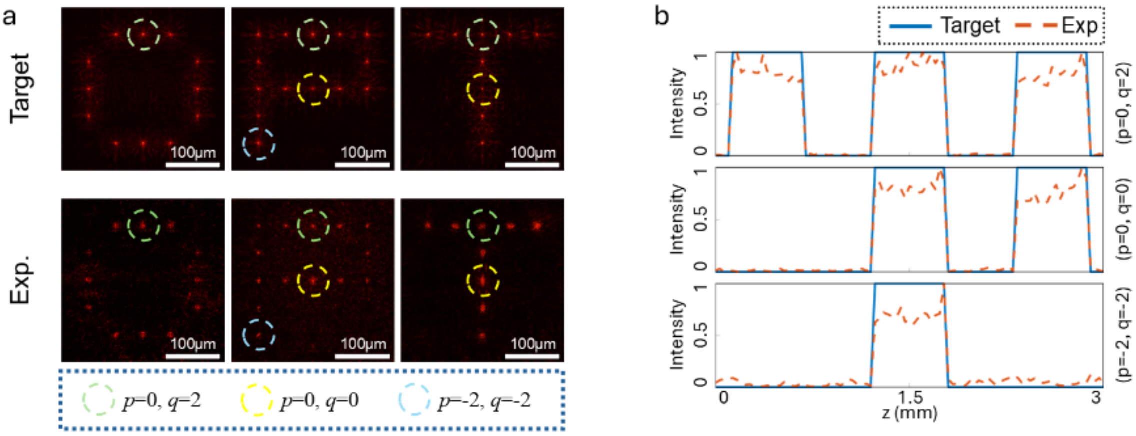

Fig. S10 | Measured and target intensity distribution of the array generated by the third metasurface, forming longitudinally varying patterns ("O," "P," and "T"). a The target and experimentally measured transverse intensity distributions at the target axial locations. Three beams corresponding to the beam coordinates ($p = 0$, $q = 0$), ($p = 0$, $q = 2$), and ($p = -2$, $q = -2$) are highlighted by the yellow, green, blue dashed circles. **b** The experimentally measured normalized intensity profiles of the three beams, compared with the target intensity lines.

Section 8: Full Stokes polarimetry along the *z*-axis

To validate the polarization control implemented by the three metasurfaces, the intensity profiles of the specific beams in the paper have been recorded at each plane along the propagation direction, filtered by different linear polarizers and circular polarizers.

To track the evolution of the polarization state, the full Stokes polarimetry is performed by placing different linear polarizers and circular polarizers before the CCD over the region z = 0-3 mm at equal intervals of 25 μm. Based on the measured 121 transverse intensity patterns at different axial locations, the intensity distributions after the three metasurfaces are obtained along the *x-z* plane after specific polarizers, as depicted in Fig. S11a, Fig. S12a, and Fig. S13a, respectively. Furthermore, the intensities of different polarization states are plotted in Fig. S11a, Fig. S12a, and Fig. S13a, with comparison against the target intensity profiles of different polarization states. The experimentally measured polarization behavior is consistent with the target function under the *x*-polarized incident beam. Considering the non-uniform longitudinal intensity profile and different losses of the polarizers, the Stokes parameters are calculated based on the six measured intensities as follows

$$S_1 = \frac{I_{0°} - I_{90°}}{I_{0°} + I_{90°}} \tag{S21a}$$

$$S_2 = \frac{I_{45°} - I_{135°}}{I_{45°} + I_{135°}} \tag{S21b}$$

$$S_3 = \frac{I_{\mathrm{RCP}} - I_{\mathrm{LCP}}}{I_{\mathrm{RCP}} + I_{\mathrm{LCP}}} \tag{S21c}$$

where $I_{0°}$, $I_{45°}$, $I_{90°}$, and $I_{135°}$ denote the on-axis intensities when the output linear polarizer is oriented at 0°, 45°, 90°, and 135°. I_{LCP} and I_{RCP} denote the on-axis intensities filtered by the left-handed and right-handed circular polarizers. It is noted that the full Stokes polarimetry of the three metasurfaces is conducted at reduced intervals of 10 μm to perform more detailed visualization of the polarization evolution within axial regions showing non-zero intensity. The longitudinal polarimetry with finer intervals is conducted over the axial region {0.64 mm ≤ z ≤ 1.16 mm} for the first metasurface, over the axial region {0.10 mm ≤ z ≤ 0.60 mm} for the second metasurface, and over the axial region {0.12 mm ≤ z ≤ 0.68 mm} for the third metasurface.

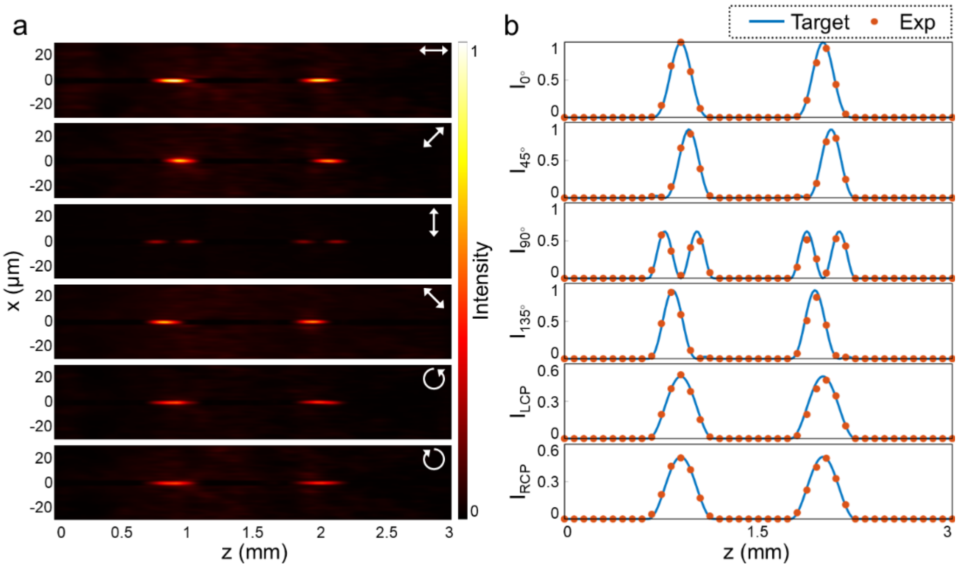

Fig. S11 | Full Stokes polarimetry of the first metasurface, showing a 180° counter-clockwise rotation around the equator of the Poincaré sphere within each region. a The experimentally measured intensity distributions around the center beam (highlighted in Fig. 3a) on the *x*-*z* plane filtered by different polarizers depicted by the white arrow. **b** The target (blue lines) and calculated (red dots) intensity lines of the beam along the *z*-axis after transmission through the corresponding polarizers depicted in (**a**).

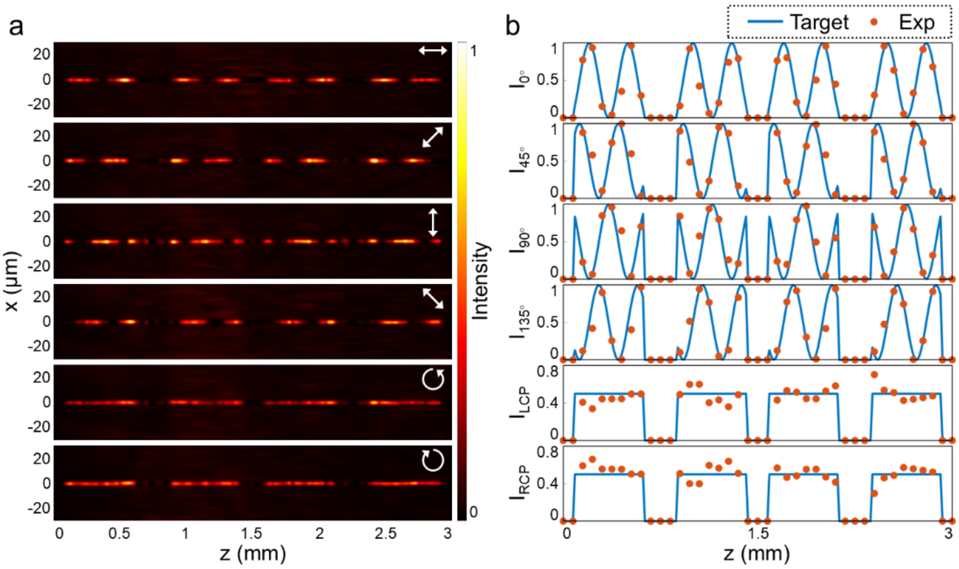

Fig. S12 | Full Stokes polarimetry of the second metasurface, showing a 360° clockwise rotation around the equator of the Poincaré sphere within each region. a The experimentally measured intensity distributions around the center beam (highlighted in Fig. 3b) on the *x*-*z* plane filtered by different polarizers depicted by the white arrow. **b** The target (blue lines) and calculated (red dots) intensity lines of the beam along the *z*-axis after transmission through the corresponding polarizers depicted in (**a**).

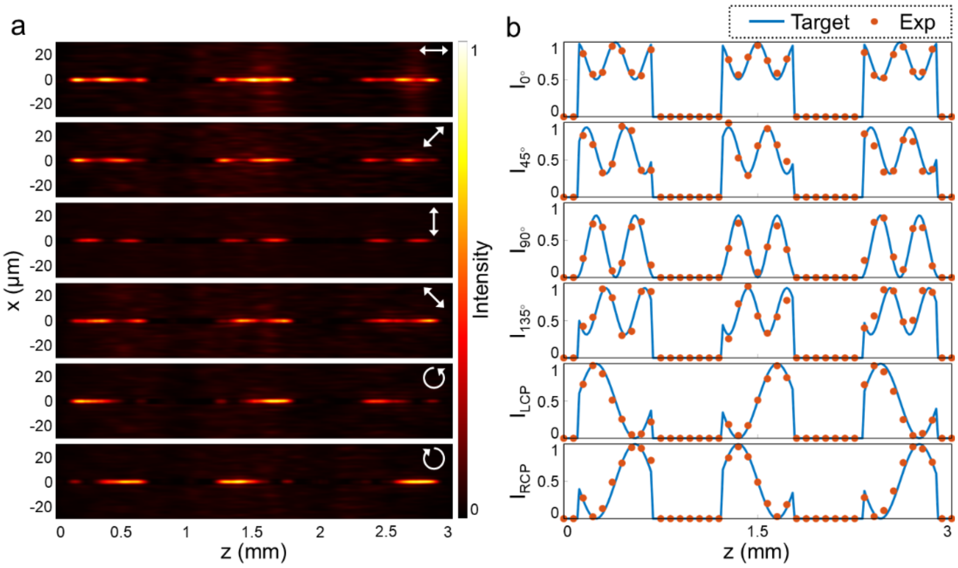

Fig. S13 | Full Stokes polarimetry of the third metasurface, showing a complex trajectory around the Poincaré sphere within each region. a The experimentally measured intensity distributions around the beam (highlighted in Fig. 4a) on the *x*-*z* plane filtered by different polarizers depicted by the white arrow. **b** The target (blue lines) and calculated (red dots) intensity lines of the beam along the *z*-axis after transmission through the corresponding polarizers depicted in (**a**).

Figures S11-S13 show that the measured on-axis polarization parameters are in agreement with the simulated predictions. The measurements confirm that the metasurfaces accurately reproduce complex longitudinal polarization dynamics with high fidelity, even along complex polarization trajectories. In addition, these results verify the metasurfaces' ability to realize continuous vectorial control along the optical axis.

Section 9: Experimental conditions for projected 3D vectorial holography

The experimental setup for 3D vectorial holography is schematically illustrated in Fig. S14. The measurements were conducted using a supercontinuum laser source (Fianium WL-SC400-4) coupled with an acousto-optic tunable filter (AOTF). A polarizer placed before the metasurface prepared the incident polarization state, while a second polarizer after the metasurface analyzed the polarization of the transmitted field. A 20× microscope objective collected the transmitted profiles and imaged the modulated beam arrays onto a CCD camera (Vihent, VTSE3S-2000). The metasurface was mounted on an automated z-translation stage (Thorlabs DRV250) to record 2D and 3D holographic images along the propagation direction.

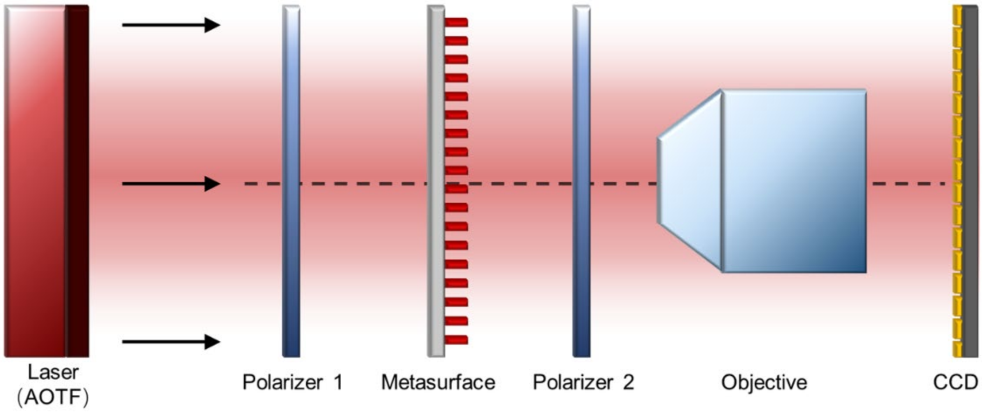

Fig. S14 | Schematic of the experimental setup for 3D vectorial holography.

Reference

[1] Preston, S.G., Watson, J.B. Generation of a harmonic quasi-continuum from beating laser fields. *J. Phys. B: At. Mol. Opt. Phys*. **31** 2247 (1998).

[2] Zamboni-Rached, M. Stationary optical wave fields with arbitrary longitudinal shape by superposing equal frequency bessel beams: frozen waves. *Opt. Express* **12**, 4001-4006 (2004).

[3] Dorrah, A.H., Bordoloi, P., de Angelis, V.S., Sarro, J.O., Ambrosio, L.A., Rached, M.Z., Capasso, F. Light sheets for continuous-depth holography and three-dimensional volumetric displays. *Nat. Photon.* **17**, 427-434 (2023).

[4] Devlin, R.C., Ambrosio, A., Rubin, N.A., Mueller, J.P.B., Capasso, F. Arbitrary spin-to-orbital angular momentum conversion of light. *Science* **358**, 896-901 (2017).

[5] Huo, P., Zhang, C., Zhu, W., Liu, M., Zhang, S., Zhang, S., Chen, L., Lezec, H.J., Agrawal, A., Lu, Y., Xu, T. Photonic spin-multiplexing metasurface for switchable spiral phase contrast imaging. *Nano Lett*. **20**, 2791-2798 (2020).

[6] Yu, N., Capasso, F. Flat optics with designer metasurfaces. *Nat. Mater.* **13**, 139-150 (2014).

[7] Liu, M., Zhu, W., Huo, P. Multifunctional metasurfaces enabled by simultaneous and independent control of phase and amplitude for orthogonal polarization states. *Light Sci. Appl*. **10**, 107 (2021).

[8] Mendoza-Yero, O., Mínguez-Vega, G., Lancis, J. Encoding complex fields by using a phase-only optical element. *Opt. Lett.* **39**, 1740-1743 (2014).

[9] Dorrah, A.H., Rubin, N.A., Zaidi, A., Tamagnone, M., Capasso, F. Metasurface optics for on-demand polarization transformations along the optical path. *Nat. Photonics* **15**, 287-296 (2021).

[10] Dorrah, A.H., Rubin, N.A., Tamagnone, M., Zaidi, A., Capasso, F. Structuring total angular momentum of light along the propagation direction with polarization-controlled meta-optics. *Nat. Commun.* **12**, 6249 (2021).
